# How alternative splicing changes the properties of plant proteins

**DOI:** 10.1017/qpb.2022.9

**Published:** 2022-07-01

**Authors:** Ivan Kashkan, Ksenia Timofeyenko, Kamil Růžička

**Affiliations:** 1 Laboratory of Hormonal Regulations in Plants, Institute of Experimental Botany, Czech Academy of Sciences, Prague, Czech Republic; 2 Functional Genomics and Proteomics of Plants, Central European Institute of Technology and National Centre for Biomolecular Research, Masaryk University, Brno 62500, Czech Republic

**Keywords:** alternative splicing, competitive inhibition, feedback loop, network motifs, plant development, RNA processing

## Abstract

Most plant primary transcripts undergo alternative splicing (AS), and its impact on protein diversity is a subject of intensive investigation. Several studies have uncovered various mechanisms of how particular protein splice isoforms operate. However, the common principles behind the AS effects on protein function in plants have rarely been surveyed. Here, on the selected examples, we highlight diverse tissue expression patterns, subcellular localization, enzymatic activities, abilities to bind other molecules and other relevant features. We describe how the protein isoforms mutually interact to underline their intriguing roles in altering the functionality of protein complexes. Moreover, we also discuss the known cases when these interactions have been placed inside the autoregulatory loops. This review is particularly intended for plant cell and developmental biologists who would like to gain inspiration on how the splice variants encoded by their genes of interest may coordinately work.

## Graphical abstract

**Figure:**
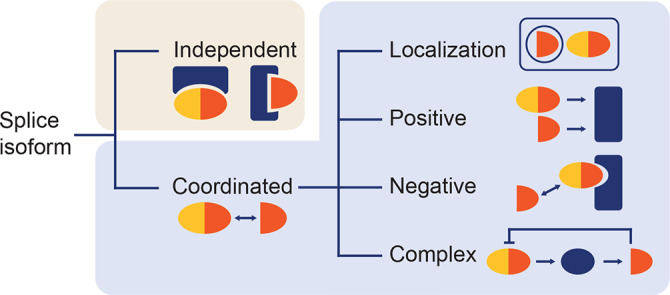

## 1. Introduction

**Fig. 1.**
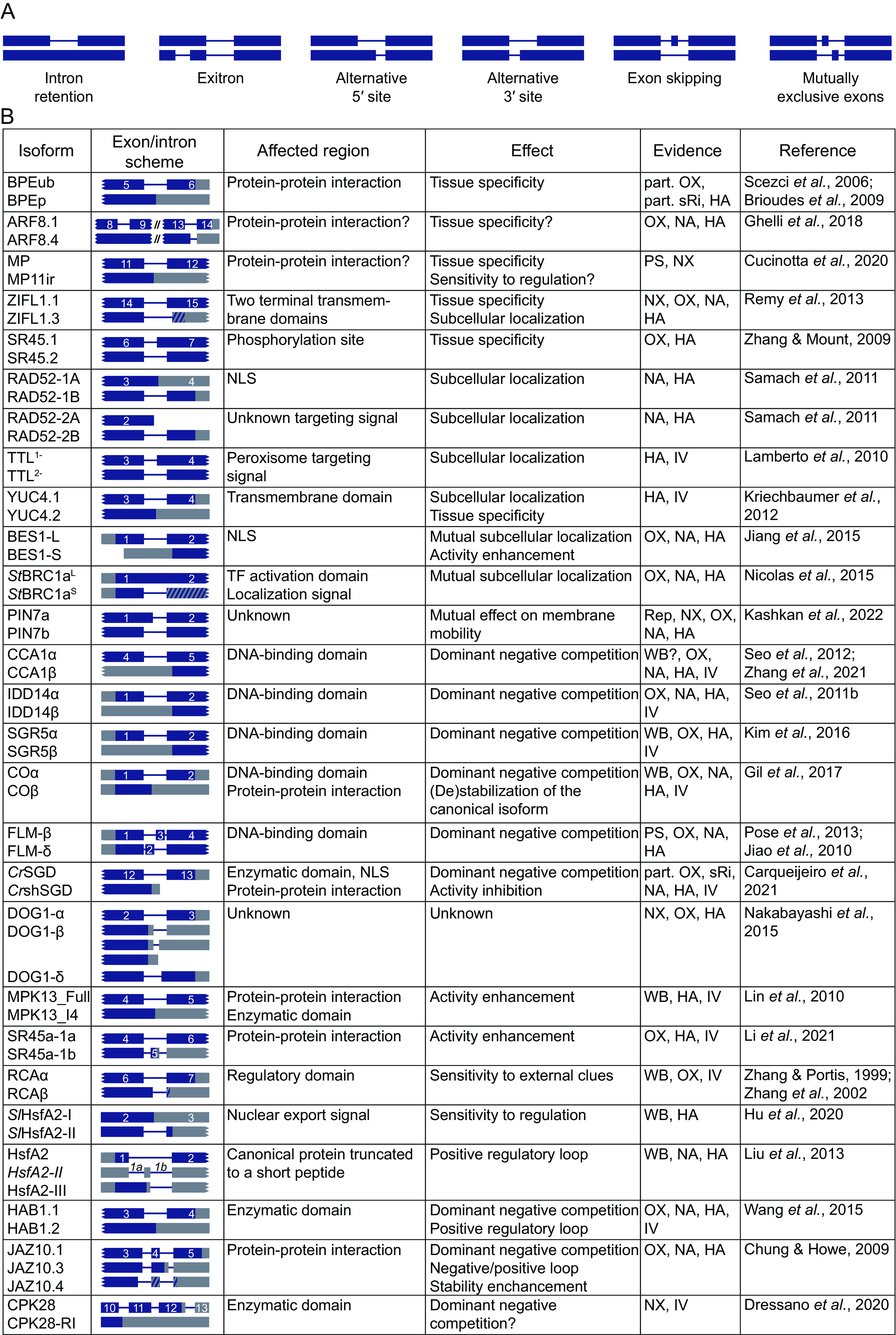
A summary of the prominent in-depth characterised AS events in plants. (a) A scheme of common AS types. (b) The list of selected in-depth characterised AS isoforms in plants. Prefixes prior to the protein symbols denote *St*, *Solanum tuberosum*, *Cr*, *Catharanthus roseus, Sl*, *Solanum lycopersicum*, no prefix indicates *Arabidopsis thaliana*. The AS diagrams show exons as boxes, introns as lines; coding sequences are coloured in blue, untranslated regions in grey, and coding regions with alternative reading frames as hatched. NLS, nuclear localization signal; TF, transcription factor. Abbreviations in the Evidence column designate the main experimental approaches supporting the existence of respective splice protein isoforms or proposed molecular models: WB, the native variants detected with western blot; Rep, AS reporter; PS, mRNA associates with polysomes; NX, phenotypes complemented by cDNA expressed under natural promoter; OX, phenotypes conferred by cDNA overexpression; sRi, transcript-specific RNAi; NA, biochemical or microscopic assays performed with cDNAs stably expressed in the native plant systems; HA, in vivo assays performed in heterologous systems, IV, in vitro assays (such as experiments with purified recombinant proteins).

In eukaryotes, almost all protein-coding primary transcripts are interrupted by introns, regions that are not translated and are removed by the process called splicing. The position and length of most introns, their 5^′^ and 3^′^ splice sites, are constitutively defined by the adjoining sequence context. However, some of them can be dynamically adjusted by the process of alternative splicing (AS) (Kelemen et al., 2013; Lee & Rio, 2015; Reddy et al., 2013; Wang & Burge, 2008). Most AS events occur in common patterns, called AS types or modes (Figure 1a). The most frequent plant AS type is intron retention (Figure 1a), which comprises ~60% of the AS events described (Chaudhary et al., 2019a; Marquez et al., 2012; Reddy et al., 2013) and includes a particular subtype called exitrons, introns with a protein-coding ability present inside exons, holding ~4% (Marquez et al., 2015). Selection of the alternative 5^′^ or 3^′^ splice sites (Figure 1a) represents ~8 and ~16%, respectively, of *Arabidopsis thaliana* events. The simultaneous change of both 5^′^ and 3^′^ splice sites, such as exon inclusion or skipping (Figure 1a), corresponds collectively to ~8% of events. The remaining AS types, such as mutual exclusion of exons, rarely occur in plants (Chaudhary et al., 2019a; Filichkin et al., 2015; Marquez et al., 2012; Martín et al., 2021).

Nearly every plant gene produces, besides the main, usually longest and most expressed, canonical transcript, one or more alternative mRNAs (Marquez et al., 2012; Zhu et al., 2017). Although a significant portion of the alternative transcripts seems to be functionally neutral (Tress et al., 2017a; 2017b), it was shown that AS affects the function of countless individual genes (Chaudhary et al., 2019b; Filichkin et al., 2015; Kelemen et al., 2013; Martín et al., 2021; Reddy et al., 2013; Staiger & Brown, 2013; Szakonyi & Duque, 2018). It has been previously insightfully reviewed how AS is carried out at the (pre-)mRNA level in plants (Reddy et al., 2013), how AS impacts the processing and function of long non-coding RNAs (Fonouni-Farde et al., 2021) and how AS patterns differ between plants and animals (Chaudhary et al., 2019b; Martín et al., 2021). The spectrum of physiological and developmental processes related to particular AS events was also discussed from various points of view (Carvalho et al., 2013; Shang et al., 2017; Staiger & Brown, 2013; Szakonyi & Duque, 2018). However, in contrast to several comprehensive reviews in the animal field (Kelemen et al., 2013; Stamm et al., 2005), the general functional consequences of AS exerted on the protein level have not been properly summarised in plants. Emphasising the cellular and developmental aspects, we have assembled the most representative and well-characterised AS events from *Arabidopsis thaliana* and other model systems to illustrate the general mechanistic principles in which the plant splice isoforms appear to coordinately work (Figure 1b).

## 2. Diverse tissue-specific roles of splice isoforms

**Fig. 2.**
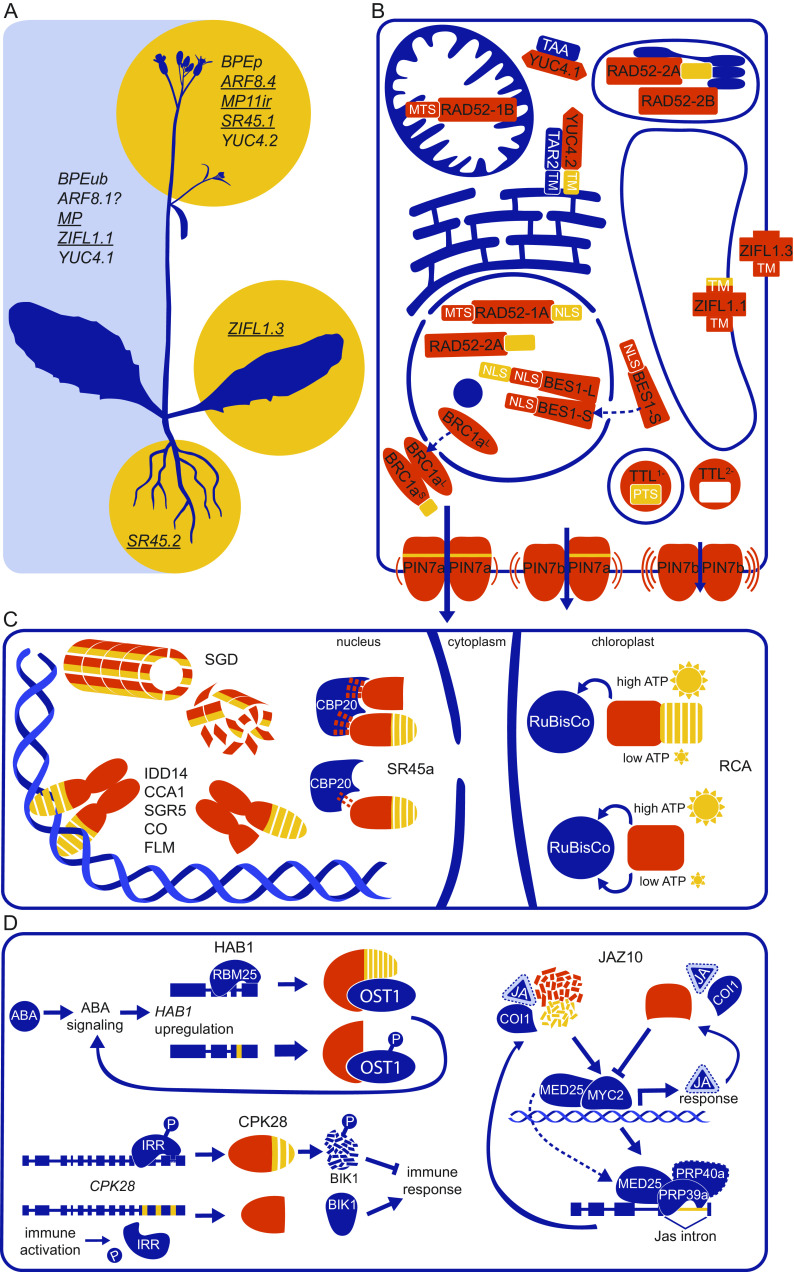
Schematic outline of main functional changes of proteins caused by AS in plants. (a) Organ-specific occurrence and functionality of representative splice isoforms. Underlined gene symbols denote the known ability to complement diverse phenotypes. While *BPEub*, *ZIFL1.1* and *YUC4.1* generally show uniform expression across organs, the *BPEp* and *YUC4.2* transcripts are enriched in flowers and *ZIFL1.3* in leaves. In contrast to *ARF8.1*, *ARF8.4* (presumably elevated in flowers) reverts only flower-specific defects of arf8 knockouts. *MP* rescues all *mp* loss-of-function phenotypes, while *MP11ir* complements only those related to ovule development. Both *SR45* variants show practically overlapping expression, but *SR45.1* reverts floral organ phenotypes, while *SR45.2* rescues root elongation defects conferred by the *sr45* loss-of-function mutation. (b) Effect of AS on the subcellular localization of splice isoforms: RAD52-1A (nucleus) and RAD52-1B (mitochondria), RAD52-2A (nucleoplasm and chloroplasts) and RAD52-2B (chloroplasts), TTL1− (peroxisomes) and TTL2− (cytosol), YUC4.1 (together with TAA in the cytosol) and YUC4.2 (together with TAR2 on the outer side of the endoplasmic reticulum), ZIFL1.1 (tonoplast) and ZIFL1.3 (plasma membrane). BES1-S is observed in the nucleus and cytoplasm, but the exclusively nuclearly localized BES1-L is able to displace BES1-S to the nucleus. On the contrary, BRC1aL is seen in the nucleus and BRC1aS in the cytoplasm, when BRC1aS directly prevents nuclear targeting of BRC1aL. PIN7a shows lower mobility within the plasma membrane (and presumably higher transporting capacity *in planta*) than PIN7b, however, both isoforms associate and can directly affect the mobility of the other protein. NLS, nuclear localization signal; MTS, mitochondrial targeting sequence; TM, transmembrane domain(s); PTS, peroxisomal targeting signal. (c) Examples of common effects of AS on protein function in plants. In case of negative interaction, alternative isoform competes with the canonical protein in the dimer and inhibits its binding to DNA (IDD14, CCA1, SGR5, CO, FLM) or abolishes the formation of the complex required for the catalytic conversion of the metabolite (SGD). Among documented positive interactions, the truncated alternative SR45a-1b isoform enhances the association of the canonical SR45a-1a variant with CBP20, a subunit of the mRNA cap-binding complex. In chloroplasts, the RCA variants coordinately function under different light conditions. (d) A scheme of the positive regulatory loop involving AS of *HAB1* in ABA signalling, both positive and negative regulatory loops implicated in AS of *JAZ10* in the jasmonate (JA) transduction cascade and the immune activation mediated by AS of CPK28. On the schemes on (b) to (d), the amino acid regions shared between both splice variants are coloured in orange and the regions modified by AS in yellow.

Transcriptomic studies have demonstrated that a substantial number of splice isoforms show differential expression in various cell types (Klepikova et al., 2016; Li et al., 2016; Martín et al., 2021). Only limited functional evidence underlies their specific expression patterns and/or distinct ability to rescue mutant loss-of-function phenotypes. The gene determining floral organ size *BIG PETAL* (*BPE*) encodes a petal-specific *BPEp* transcript with the last intron retained, in addition to the canonical *BPEub* with a uniform expression in all organs (Figures 1b and 2a) (Szécsi et al., 2006). The *C*-terminal sequence exclusively encoded by *BPEp* is required for the interaction with the AUXIN RESPONSE FACTOR 8 (ARF8), a component of auxin signalling cascade involved in floral organ development. The *arf8* loss-of-function mutants phenocopy the petal defects of the *bpe* knockout mutants. It was therefore assumed that BPEp, in contrast to the *bona fide* BPEub protein, is required for petal development (Varaud et al., 2011; Zhang et al., 2020). *ARF8* seems to undergo tissue-specific AS as well. The relative levels of the alternative *ARF8.4* variant (showing the in-frame retention of the eighth intron along with the alternative 5^′^ site in the last intron and premature stop codon) were recognised as elevated in flowers (Figures 1b and 2a). The overexpression of the *ARF8.4* cDNA, but not other *ARF8* isoforms, reverts stamen elongation defects associated with the *arf8* knockout mutation (Ghelli et al., 2018). The essential regulator of the early embryogenesis *AUXIN RESPONSE FACTOR 5*/*MONOPTEROS* (*ARF5*/*MP*) produces an alternative transcript denoted *MP11ir* with the last (eleventh) intron retained, leading to the protein truncation (Figure 1b). *MP11ir* cDNA complements ovule-specific defects conferred by the *mp/arf5* loss-of-function mutation, in contrast to the canonical *ARF5* variant that also rescues the remaining, post-embryonic *mp/arf5* defects. Both isoforms show rather similar expression pattern, but it was speculated that the truncated MP11ir protein might be instrumental for an auxin-independent activation of the downstream transcriptional pathways in the early ovule development (Figure 2a; Cucinotta et al., 2021).

The overexpression of the canonical transcript encoding the membrane transporter ZINC-INDUCED FACILITATOR-LIKE 1 (*ZIFL1.1*, expressed ubiquitously) rescues several auxin-related defects associated with the *zifl1* knockout mutation. Nevertheless, the predominantly leaf-specific *ZIFL1.3* variant (originating from the 3^′^ alternative splice site in the 14th intron) (Figures 1b and 2a) reverts the subset of phenotypes linked with the abscisic acid (ABA) and drought response (Remy et al., 2013). Similarly, the gene encoding the regulator of AS called SERIN-ARGININE-RICH PROTEIN 45 (SR45) is regulated by the choice of the alternative 3^′^ site in the sixth intron, which removes a short amino acid motif with a critical phosphorylation site (Figure 1b). Both transcripts are expressed across most tissues at comparable levels. However, only the full-length *SR45.1* variant can rescue the narrow petal phenotypes observed in the *sr45* loss-of-function mutants. *SR45.2*, on the contrary, complements exclusively their root elongation defects (Figure 2a) (Zhang & Mount, 2009; Zhang et al., 2014).

AS of *ARF5*, *ARF8* and *BPE* appears to modify the respective targets in a cell-specific manner to tune the tissue or organ identity. In the case of *ZIFL1* and *SR45*, AS intriguingly changes the fundamental functional outcomes of the resulting proteins, and it would be exciting to explore further the mechanistic principles underlying these findings.

## 3. Differential subcellular localization

One of the most noticeable features of AS is the capability to change the subcellular localization of the protein (Figure 2b). The *RADIATION SENSITIVE 52* (*RAD52-1)* gene, required for the homology-dependent DNA double-strand break repair, encodes a *RAD52-1A* isoform with the last intron retained (Figure 1b). RAD52-1A was shown to be localized to the nucleoplasm, while the full-length RAD52-1B isoform is targeted to mitochondria (Figure 2b). The concurrent event in the near *RAD52-2* paralog gives rise to the RAD52-2A variant, present in both nucleoplasm and chloroplast, and RAD52-2B, detected exclusively in chloroplasts (Figure 2b; Samach et al., 2011). Thus, AS controls the delivery of the RAD52 proteins (and DNA repair) between semiautonomous organelles and nuclei. TRANSTHYRETIN-LIKE (TTL), a protein required for the synthesis of allantoin, is modified by the choice of the alternative 3^′^ site of the last intron (Figure 1b). The resulting isoforms, TTL^1−^ and TTL^2−^, are localized in peroxisomes and cytoplasm, respectively (Figure 2b) (Lamberto et al., 2010). In addition, the full-length ZIFL1.1 protein (see above) is localized to the tonoplast, while the truncated isoform ZIFL1.3 is targeted to the plasma membrane (Figure 2b; Remy et al., 2013).

*YUCCA 4* (*YUC4*) encodes a rate-limiting factor required for auxin biosynthesis. The full-length YUC4.1 protein is localized in the cytosol. The alternative (and flower specific) YUC4.2 variant originates from the transcript showing retention of the last intron (Figure 1b). This region encodes a transmembrane domain that holds the YUC4.2 protein at the cytosolic side of the endoplasmic reticulum (Figure 2b). Both isoforms are catalytically active (Kriechbaumer et al., 2012). Later, these observations were placed into a broader context. TRYPTOPHAN AMINOTRANSFERASE OF ARABIDOPSIS 1 (TAA1) and its paralog TRYPTOPHAN AMINOTRANSFERASE RELATED 2 (TAR2) show metabolic activity directly upstream of YUC and reside in the cytosol and on the endoplasmic reticulum, respectively. Moreover, other YUC paralogs (unprocessed by AS) as well localize either in the cytosol or on the endoplasmic reticulum (Figure 2b). Hence, TAA1/TAR2 and the YUC4 isoforms closely associate in both compartments to convert tryptophan to auxin in the formed metabolons (Hrtyan et al., 2015; Kriechbaumer et al., 2017).

The spatial non-overlapping detachment of the splice isoforms inside the cell apparently indicates that they function independently. Although it seems that there can be cases where AS leads, for example, to deactivation of the protein by its deposition in a different compartment (see also Jiang et al., 2015 and Nicolas et al., 2015 below), the RAD52-1/2 and YUC4 AS outcomes resemble products of separate genes, analogous to gene duplication.

## 4. Mutually dependent subcellular distribution

In contrast to the examples where protein isoforms act likely independently, a large part of studies reveals that splice isoforms mutually affect each other’s function. For instance, they can coordinately influence their subcellular localization by direct molecular interaction (Figure 2b). The longer BES1-L variant of the transcriptional factor BES1 (BRI1 EMS SUPPRESSOR 1), involved in brassinosteroid signalling, contains two bipartite nuclear localization signals (NLS), which promote retention of the protein in the nucleus. The shorter BES1-S isoform (designated as canonical due to the sequence conservation) with the alternative transcription initiation codon in the second exon lacks the *N*-terminal part, including the first NLS, and is observed in both nucleus and cytoplasm (Figure 1b). When BES1-S was co-expressed together with BES1-L, it was detected only in the nucleus, probably due to the dimerisation with BES1-L (Figure 2b). The BES1-mediated relocation to the nucleus was also shown for another component of brassinosteroid signalling, BRASSINAZOLE-RESISTANT 1 (BZR1), which otherwise displays dual cytoplasmic and nuclear localization as well. Both BES1-L and BES1-S isoforms are probably functional. However, the overexpression of BES1-L, but not BES1-S, leads to the phenotypes associated with brassinosteroid (Jiang et al., 2015). The gene *BRANCHED1a* (*BRC1a*) codes for a TCP (bHLH) transcription factor, which is processed into two isoforms in the *Solanum* genus. The nuclear-localized canonical BRC1a^L^ variant with the retained in-frame first intron (more potent at inducing ectopic defects when overexpressed) carries a transcription activation domain on the *C*-terminus (Figure 1b). Splicing of the first intron in the shorter BRC1a^S^ transcript leads to the replacement of the activation domain by a frame-shifted amino acid sequence and prevents the nuclear targeting of BRC1a^S^ from the cytoplasm. The co-expression of BRC1a^L^ and BRC1a^S^ results in their dimerisation and a partial shift of BRC1a^L^ to the cytosol, along with the decreased ability to induce the reporter-monitored BRC1a-dependent transcription (Figure 2b; Nicolas et al., 2015).

PIN7 (PIN-FORMED 7), an auxin efflux carrier polarly localized on the plasma membrane, is encoded by two major transcripts. The shorter *PIN7b* is generated by the choice of an alternative 5^′^ splice site in the first intron (Figure 1b). The resulting protein lacks a 4-amino acid stretch inside the large internal hydrophilic loop (Hrtyan et al., 2015). The longer *PIN7a* variant, expressed under native promoter, rescues the tropic bending responses and other defects associated with the *PIN7* locus, even leading to exaggerated phenotypes. In contrast, *PIN7b* is almost inactive when expressed alone. Both isoforms show the comparable capability of transporting auxin in a heterologous system and similar subcellular localization in the native tissues. However, tracking with the fluorescence recovery after photobleaching revealed that PIN7a shows lower lateral mobility within the plasma membrane than PIN7b. Moreover, PIN7a and PIN7b form homo- and heterodimers and show the rates of lateral mobility dropping closer to intermediate values when co-expressed (Figure 2b). Consistently, *PIN7b* reverts the exaggerated tropic response conferred by *PIN7a*, phenocopying that of the wild-type *PIN7* allele (Kashkan et al., 2020; 2021).

On the outlined examples, the localization overlap marks the likely area where the splice isoforms interact and influence each other’s presence in the given spot. The external cues can tune the resulting activity of the AS products population in the cell. For example, the *BRC1a* transcript ratios can change following various environmental stimuli (light conditions, decapitation, hormonal treatment) (Nicolas et al., 2015). Similarly, the levels of *PIN7b* or both *BES1* isoforms can be changed by the application of the respective hormone, likely compensating the response to the growth regulator (Jiang et al., 2015; Kashkan et al., 2020; 2021).

## 5. Competitive inhibitory effects

The truncated alternative isoforms commonly show the ability to interfere with the canonical proteins. This has been particularly explored on transcription factors, which tend to form homo- or heterodimers (Seo et al., 2011a). CIRCADIAN CLOCK-ASSOCIATED 1 (CCA1) is a transcriptional factor involved in circadian regulation and cold acclimation. In contrast to the full-length CCA1α, the alternative CCA1β isoform, arising from the alternative initiation codon in the fourth exon, lacks the MYB-type DNA-binding motif (Figure 1b). This can abolish the homodimerisation of CCA1α (and also outcompetes the CCA1α paralog LATE ELONGATED HYPOCOTYL (LHY), dimerising with CCA1α as well), preventing it from binding to the promoters of selected downstream target genes (Figure 2c). Accordingly, the simultaneous presence of the *35S:CCA1β* transgene can suppress the *35S:CCA1α* overexpression phenotypes. Moreover, the overexpression of a single *CCA1α* or *CCA1β* shows opposite effects on the transcription of internal circadian rhythm markers and on the survival rates during cold acclimation (Seo et al., 2012).

IDD14 (INDERMINATE DOMAIN 14) is a bHLH transcription factor involved in various morphogenetic processes. Analogously to *CCA1*, *IDD14* encodes an alternative IDD14β isoform arising from the retention of the first intron and alternative initiation codon in the second exon (Figure 1b). Due to the missing DNA binding domain at the *N*-terminus, IDD14β is inactive. However, it heterodimerises with the canonical IDD14α isoform and inhibits its ability to bind the promoter of the downstream target genes, including *QQS* (*QUA-QUINE STARCH*), a factor responsible for the starch degradation (Figure 2c). During cold stress, the proportion of IDD14β increases and the *QQS* expression is reduced, leading to the elevated starch content (a general indicator of cold acclimation), and these effects can be reverted by the *IDD14α* overexpression (Seo et al., 2011b). Furthermore, an AS-mediated mechanism of heat-mediated shoot tropic response was proposed by Kim et al. (2016). A close paralog of *IDD14*, *SHOOT GRAVITROPISM 5* (*SGR5* or *IDD15*) shows a virtually identical isoform interaction scheme, including analogous AS type (Figures 1b and 2c). The *sgr5* knockouts show defects in shoot gravitropism, and this phenotype can indeed be rescued by the overexpression of the canonical SGR5α isoform at ambient temperature. The expression of the inhibitory SGR5β isoform increases with growing temperature. In accord with the proposed model, wild type shows reduced shoot gravitropism at increased temperature, while the shoots of the *sgr5* knockouts overexpressing the sole *SGR5α* isoform display practically normal gravitropic bending response even under elevated heat conditions (Kim et al., 2016).

CONSTANS or B-BOX DOMAIN PROTEIN 1 (CO or BBX1) is a transcription factor that regulates photoperiodic flowering by controlling the integrator gene *FLOWERING LOCUS T* (*FT*). Due to the retention of the only intron and premature stop codon presence, the alternative COβ variant lacks the *C*-terminal CCT domain responsible for binding DNA (and several other proteins interacting with FT) (Figure 1b). COβ heterodimerises with the full-length COα isoform and prevents it from binding DNA (Figure 2c). Moreover, the presence of COβ in the dimer appears to promote the COα degradation by HOS1 (HIGH EXPRESSION OF OSMOTICALLY RESPONSIVE GENES 1) and COP1 (CONSTITUTIVE PHOTOMORPHOGENIC 1), CO-destabilising ubiquitin E3 ligases, but inhibits its binding to the CO-stabilising E3 ubiquitin ligase FKF1 (F-BOX 1). Thus, the overall COα levels seem to be negatively regulated during the night (HOS1) or in the morning (COP1). In the late afternoon, COα can be temporarily preserved (FKF1), being even protected itself from binding to COβ. The diurnally elevated levels of COα can thereby promote flowering during the long day conditions (Gil et al., 2017). A similar functional model has also recently been hypothesised for the CO ortholog (Huang et al., 2022; Jiao & Meyerowitz, 2010; Job et al., 2018).

Ultimately, a similar isoform interplay was shown for *FLOWERING LOCUS M* (*FLM*, see the scheme of mutual exon exclusion on Figure 1b), a MADS-box transcription factor involved in the regulation of flowering at increased temperature (Lee et al., 2013; Posé et al., 2013), and parallelised by the FLM paralog MADS AFFECTING FLOWERING 2 (MAF2) (Airoldi et al., 2015). Among splice variants, FLM-δ does not bind DNA but competes with the functional FLM-β isoform for the interaction with the SHORT VEGETATIVE PHASE (SVP) protein, a co-repressor of flowering (Figure 2c). While the levels of FLM-β decrease with the growing temperature, the amounts of FLM-δ rise, releasing the block on the downstream transcripts required for early flowering and the downstream developmental response (Lee et al., 2013; Posé et al., 2013). The whole mechanism is perhaps more complicated. Further research revealed that a sole decrease of the FLM-β levels is sufficient to induce early flowering (Capovilla et al., 2017; John et al., 2021; Lutz et al., 2015; 2017; Sureshkumar et al., 2016), and the *FLM-β* amounts at the elevated temperature appear to be lowered by the preferential production of other transcripts that are subsequently degraded by non-sense mediated decay (NMD) (Sureshkumar et al., 2016).

Besides interfering with the DNA-binding activity, the dominant-negative alternative isoforms were demonstrated to abolish the catalytic activity of the canonical variants of metabolic enzymes. STRICTOSIDINE β-d-GLUCOSIDASE (SGD) is involved in the synthesis of the cytotoxic monoterpene indole alkaloids in *Catharanthus roseus*. The alternative variant shSGD lacks a large part of the *C*-terminal sequence, including NLS, resulting from the retention of the last intron and a premature stop codon (Figure 1b). In contrast to the canonical SGD variant, shSGD is catalytically inactive and unable to self-interact. However, it can heterodimerise with SGD and even disrupts the high-molecular complexes formed by SGD in vitro (Figure 2c). shSGD thereby directly inhibits the enzymatic activity of SGD and affects the synthesis of the relevant monoterpene indole alkaloids *in planta*. In contrast to the nucleus-resided SGD variant, shSGD shows a dual nuclear and cytosolic localization. In the bimolecular fluorescence complementation interaction assays, it binds also THAS1, another nuclear enzyme involved in further steps of the alkaloid synthesis which normally complexes with SGD. Moreover, shSGD can recruit THAS1 to the cytosol, even when co-expressed with the canonical SGD variant (Carqueijeiro et al., 2021).

A high number of studies illustrate how minor truncated isoforms can interfere with the activity of the full-length proteins. This mode of action seems to be common in most eukaryotes (Jangi & Sharp, 2014; Seo et al., 2011a). Removal of protein domains by AS typically reduces the number of interaction partners at least in half in animal systems (Rodriguez et al., 2020; Yang et al., 2016). Mathematical models of regulatory network motifs indicate that gene expression systems containing dominant-negative factors (here alternative isoforms) show faster response times following the signal stimulus. It can thereby represent a potent adaptation to the changing external or developmental cues (Alon, 2007; Jangi & Sharp, 2014).

## 6. Various manners of cooperative action of splice isoforms

Occasionally, the interaction of the canonical and alternative variant(s) can lead to a complex functional interaction in the expressed isoform assemblage. AS of *DOG1* (*DELAY OF GERMINATION 1*), a regulator of seed dormancy, leads to five mRNAs, eventually producing three proteins. If overexpressed, they complement the *dog1* loss-of-function phenotypes. However, when these cDNAs were expressed under the natural promoter alone, the resulting proteins were degraded rapidly, failing to restore the *dog1* dormancy defects of the mutant entirely (Nakabayashi et al., 2015), or at least moderately (Cyrek et al., 2016). The expression of two or more isoforms stabilises by unknown mechanism the subsequent DOG1 accumulation in the nucleus and can rescue the *dog1* knockout phenotypes (Nakabayashi et al., 2015).

The gene encoding the *MITOGEN-ACTIVATED PROTEIN KINASE* 13 (*MPK13*) gives rise to a truncated alternative transcript *MPK13_I4* with the fourth intron retained (Figure 1b). In contrast to the canonical MPK13_Full isoform, MPK13_I4 lacks a part of the kinase domain. MPK13_I4 alone does not show the typical (auto)phosphorylation activity, nor the ability to interact with the MKK6 (MITOGEN-ACTIVATED PROTEIN KINASE 6) acting upstream of MPK13. However, adding the recombinant MPK13_I4 protein into the in vitro reaction mixture enhances the activation of MPK13_Full by MKK6 (Lin et al., 2010). Similarly, the alternative truncated isoform SR45a-1b (different from the SR45 protein above), resulting from the cryptic fifth exon, cannot interact with another core spliceosome component U1-70K due to the lack of the essential *C*-terminal RNA-binding RS domain. However, it remains partially functional in the salt-stress response linked with the SR45a protein and enhances the formation of the complex of the full-length SR45a-1a isoform and the CBP20 cap-binding protein, along with the regulation of AS of numerous salt-stress related genes (Li et al., 2021).

The first step of the chloroplast fixation of CO_2_ in the Calvin cycle is co-regulated by AS of nuclearly encoded *RUBISCO ACTIVASE* (*RCA*). The longer *RCAα* (or *RCA_L_*) and the shorter *RCAβ* (*RCA_S_*) transcripts differ in the choice of the 5^′^ splice site in the last intron, leading to the frameshift and protein truncation (Figure 1b; Werneke et al., 1989). In multiple species, including *Arabidopsis thaliana*, both proteins activate Rubisco in vitro. However, the truncated RCAβ lacks cysteine residues required for the perception of fluctuating ADP levels (or of changed redox conditions) and bypasses the feedback loop reacting on the shortage of ATP occurring at the decreased light intensities (Figure 2c; Shen et al., 1991; Zhang & Portis, 1999). The joint action of both isoforms is a part of adaptation to light conditions: the lines harboring RCAα alone are unable to reach the wild-type rates of Rubisco activation under saturating light conditions. In contrast, the lines carrying exclusively RCAβ show the steadily elevated Rubisco activity, regardless of high- or low-light conditions used. Only the lines containing both RCAα and RCAβ in the *rca* knockout mutant background display the Rubisco activation dynamics similar to wild type (Zhang et al., 2002). It was also shown that the expression levels of both isoforms can be regulated by altering the external temperature or during heat acclimation, and that both isoforms seem to be responsible for different photosynthetic activities under (heat) stress conditions (reviewed in Carvalho et al., 2013).

Hu et al. (2020) performed a set of protoplast assays to explore the function of two out of seven variants of the HEAT SHOCK TRANSCRIPTIONAL FACTOR A2 (HsfA2) in tomato. HsfA2-I is the longest isoform. It possesses both nuclear export and NLSs and shuttles between the nucleus and cytoplasm. *HsfA2-II* carries a cryptic intron towards its 3^′^ terminus, which removes the *C*-terminal nuclear export signal (Figure 1b). The HsfA2-II protein exhibits a predominantly nuclear localization and decreased protein stability. In contrast to HsfA2-I, HsfA2-II shows a limited ability to interact with the Hsp17.4-CII (HEAT SHOCK PROTEIN 17.4-CII), required for its deposition in the heat shock granules. However, both isoforms can induce transcription of the heat-shock responsive genes. The comparison of the allele polymorphisms further supported the scheme that HsfA2-I can be stored in the heat stress granules over a longer time period and re-used in case of repeated heat exposure, while HsfA2-II can be rather involved in the immediate heat-stress response (Hu et al., 2020).

Several models of how splice variants may coordinately interact have been proposed. The mechanism propounded for the DOG1 protein variants can draw up a situation when multiple isoforms are synergistically required for the correct activity of the resulting protein population. Such systems act as a sign-sensitive filter, which creates a response delay and buffers irregular (stochastic) weak signals, responding only to pronounced stimuli (Alon, 2007). Systems containing positive autoregulation, such as MPK13 and SR45a, show slower response time or result in an increased signal variability within the examined cell population, depending on the strength of the input signal (Alon, 2007; Jangi & Sharp, 2014). The elementary functions of the RCA and HsfA2 are equivalent, but they are adapted to different external cues. This can improve the system robustness in the changing conditions (Alon, 2007; Jangi & Sharp, 2014). Moreover, the evolutional analysis revealed that the two RCA proteins are encoded by separate genes in some species, conceptually similar to some variants with diverse subcellular localization discussed above (Huang et al., 2022; Nagarajan & Gill, 2018).

## 7. Complex autoregulatory circuits tuning splice isoform activity

Several studies uncovered that the splice isoforms participate in positive and negative regulatory loops. These findings integrate the previously outlined basic schemes and illustrate the envisaged complexity of the AS-mediated pathways. The main *HsfA2-I* isoform of *Arabidopsis thaliana* contains only two exons, in contrast to the situation in tomato (see above). A mild heat stress under 37°C activates a short cryptic exon splitting the only canonical intron 1 into intron 1a and 1b (Figure 1b). The intervening short sequence introduces a premature stop codon, and the resulting *HsfA2-II* transcript is eventually not translated, being probably subjected to NMD (Sugio et al., 2009). Under a severe temperature pulse, up to 45°C, the 1a intron incorporates into mRNA as well and gives rise to a leucine-rich motif in the nascent amino acid sequence within the translated *HsfA2-III* isoform (Figure 1b). The resulting short protein lacks the dimerisation and the *C*-terminal transactivation domain. However, it contains a partially truncated DNA-binding motif and can bind the heat-shock elements in its own promoter, further promoting *HsfA2* expression under extreme heat conditions. *HsfA2* thus represents an example of a positive autoregulatory loop (Liu et al., 2013).

Another positive autoregulatory loop was described for HAB1.2, a truncated isoform resulting from the retention of the last intron of the gene coding for the HAB1 (HYPERSENSITIVE TO ABA 1) phosphatase, a negative regulator of the ABA signalling pathway (Figure 1b). HAB1.2 is ABA inducible and binds the downstream protein kinase OST1 (OPEN STOMATA 1), a positive regulator of ABA response, without the ability to dephosphorylate it. The overexpression of the canonical *HAB1.1* transcript in the *hab1-1* knockouts leads to the increased resistance to ABA, while *HAB1.2* confers the hypersensitivity even exceeding the *hab1-1* phenotypes. RBM25 (RNA-BINDING PROTEIN 25), a core regulator of AS, directly binds the last intron of the *HAB1* transcript (Figure 2d). Accordingly, the *rbm25* loss-of-function mutants show hypersensitivity to ABA and enhanced intron retention rates in several genes, particularly in *HAB1*. Hence, it was proposed that ABA increases the *HAB1.2*/*HAB1.1* expression ratio with the contribution of RBM25 to keep the ABA signal transduction active (Wang et al., 2015; Zhan et al., 2015).

Numerous RNA-binding factors show the ability to bind their own transcript to induce AS, leading to the production of the variants that apparently remain untranslated, thus turning off their own expression (Schöning et al., 2008; Hartmann et al., 2018; Quesada et al., 2003). A complex negative auto-regulatory loop, occurring arguably at the protein level, was described for JAZ10 (JASMONATE ZIM DOMAIN PROTEIN 10), a major transcriptional repressor of the nuclear-located jasmonate signalling pathway (Figure 2d). The *JAZ10.3* and *JAZ10.4* variants are produced by the choice of the alternative 5^′^ site in the last and, respectively, second last intron of the *JAZ10* primary transcript (Figure 1b). This results in the partial (JAZ10.3) or complete (JAZ10.4) removal of the conserved Jas motif that under normal conditions binds the MYC2 bHLH transcriptional factors to repress jasmonate-dependent signalling. The Jas motif is recognised by COI1 (CORONATINE INSENSITIVE 1), a F-box protein serving as jasmonate co-receptor, which in the presence of the hormone targets JAZ10 for ubiquitination, leading to the derepression of the MYC2 factors and triggering the downstream response. JAZ10.4 is practically unable to interact with COI1, while the ability of JAZ10.3 to bind COI1 is impaired only partially. Thus, both JAZ10.3 and JAZ10.4 repressors show increased stability following the jasmonate treatment (Chung & Howe, 2009).

Interestingly, the crystallographic studies revealed that JAZ10.4 could bind MYC2 transcription factors even stronger than major JAZ10.1 due to the presence of a cryptic MYC2-interacting domain (CMID) located on its *N*-terminus (Zhang et al., 2017). Moreover, while the levels of JAZ10.1 can gradually drop due to the COI1-mediated degradation, the *JAZ10.4* expression is induced by the jasmonate treatment. Hence, MYC2 factors, initially derepressed by degradation of JAZ10.1, are bound by JAZ10.4 through the CMID domain and return to the repressed state, attenuating the excessive jasmonate response by a negative feedback loop (Moreno et al., 2013; Zhang et al., 2017; Figure 2d).

MEDIATOR TRANSCRIPTIONAL COACTIVATOR 25 (MED25), a part of the multimeric Mediator complex, directly binds MYC2 to promote the jasmonate response by recruiting RNA polymerase II to the promoters of the jasmonate-responsive genes (Chung & Howe, 2009; Howe et al., 2018; Yan et al., 2007; Zhang et al., 2017). Upon the MYC2 repression by the JAZ proteins, MED25 associates with the jasmonate-inducible PRP39a and PRP40a (PRE-MRNA-PROCESSING FACTOR39a and 40a) splicing factors. They together interact with the *JAZ10* primary transcript and shift AS towards the production of the canonical *JAZ10.1* mRNA, preventing the excessive desensitisation of jasmonate signalling (Wu et al., 2020). Altogether, it seems that AS of *JAZ10* can be tuned by both positive and negative feedback loops (Figure 2d).

A thorough experimental effort unraveled the mechanisms accompanying the AS of CALCIUM-DEPENDENT PROTEIN KINASE 28 (CPK28), a negative regulator of plant innate immunity. CPK28 phosphorylates a key positive regulator of plant immunity BIK1 (BOTRYTIS-INDUCED KINASE 1), causing its degradation and attenuation of the downstream immune response. In the absence of the signal associated with the pathogen infection, IMMUNOREGULATORY RNA-BINDING PROTEIN (IRR) is phosphorylated and binds the *CPK28* pre-mRNA, activating the preferential splicing of the long, fully functional CPK28 isoform to keep the immunogenic pathways inactive (Figure 1b). Following the immune activation by plant elicitor peptides (Peps), dephosphorylated IRR dissociates from the *CPK28* primary transcript, which leads to the preferential expression of the *CPK28-RI* mRNA with the last three introns retained. CPK28-RI lacks two Ca^2+^-binding EF-hand domains and shows a severely impaired kinase activity, failing to phosphorylate BIK1. That leads, in turn, to the stabilisation of BIK1 and derepression of the Peps-triggered immune response (Figure 2d) (Dressano et al., 2020).

## 8. Limits of our knowledge, future directions

Despite the relatively limited number of elaborated studies, several molecular models have been proposed to manifest the diverse roles of splice isoforms in plants. In essence, they can operate either independently or in a joint manner. Independently acting proteins tend to show different tissue-specific expression or subcellular localization. Here, AS can fundamentally change protein roles (represented by ZIFL1 and SR45) or in effect substitute gene duplication (RAD51, RAD52, YUC4, also RCA). Interaction of splice isoforms, in its turn, represents a level of functional regulation, repressing or modifying the activity of the final protein product(s). The splice isoforms can sometimes mutually influence their subcellular localization (BES1, BRC1a, PIN7). Various examples of positive or coordinated modes of action have been also shown (DOG1, MPK13, SR45a, RCA). Nonetheless, a high number of reports demonstrated functional mechanisms involving dominant-negative (competitive) interaction, particularly on DNA-binding transcription factors (CCA1, IDD14, SGR5, CO, FLM) and also on a metabolic enzyme (SGD). That may perhaps reflect the high rate of intron retention observed in plants (Marquez et al., 2012). Functionally, it was associated with a rapid reaction to various stimuli. Accordingly, it was revealed that the negative (auto)regulation is the most common network motif in the organismal signalling pathways (Alon, 2007; Jangi & Sharp, 2014; Lee et al., 2002). The feedback loops with negative autoregulation (reported for HsfA2, HAB1, CPK28, and particularly for JAZ10) are not much explored, but they likely accompany many or most of the proposed interaction modes. Works of Shikata et al. (2014) or Huang et al. (2022) showed a possible large-scale biology-based direction, how to identify such loops and to integrate them among other signalling pathways.

Surprisingly, the extent to which AS produces the physiologically relevant protein-coding transcripts remains highly debated (Blencowe, 2017; Tress et al., 2017a; 2017b). Depending on the experimental approach (e.g., transcript association with polysomes, proteomics or evolutional conservation), the predicted share of functionally relevant AS events ranges from far negligible amounts (Abascal et al., 2015; Tress et al., 2017a) to almost half of all expressed transcripts in human and *Arabidopsis thaliana* (Reixachs-Solé et al., 2020; Weatheritt et al., 2016; Yu et al., 2016). Additionally, many AS events can be specifically activated following specific external stimuli or in small cell groups within particular tissues (Kelemen et al., 2013; Martín et al., 2021; Reddy et al., 2013; Rodriguez et al., 2020). It can be thereby often challenging to confirm their exact functional context in the controlled laboratory condition.

Moreover, a few additional methodological issues have been pointed out, particularly for plant model systems. Current gene schemes, including their protein-coding regions, are based mainly on algorithmic predictions. Hence, many annotated transcripts may not immediately code for proteins, exerting their role at the RNA level. These can also be intermediary products from various stages of mRNA maturation, subject of NMD, or even experimental artefacts. Furthermore, the actual open reading frames can largely differ from the predicted ones as well (Brown et al., 2015). In this context, it is, for example, discussed whether the *CCA1* transcripts indeed code for authentic proteins (Brown et al., 2015; Seo et al., 2012; Zhang et al., 2021). We have summarised the experimental evidence underlying the natural presence of the outlined protein isoforms, reinforcing the proposed molecular models (Figure 1b). Ideally, the immunoblotting (and the complementation test) has been suggested as solid proof. Additionally, perhaps AS reporter, the association of the transcript with the polyribosome, individually with other indirect data, can serve as a piece of good evidence supporting the authenticity of the protein variant (Brown et al., 2015; Chaudhary et al., 2019a; Kanno et al., 2018; Kashkan et al., 2020). Hence, the combined high- and low-scale experimental effort may continuously clear out the current mysteries of the physiological relevance and the most common *modi operandi* of AS.

## Data Availability

No new data or code are presented in this paper.
