# Supplementary material for: How alternative splicing changes the properties of plant proteins
Source: Quant Plant Biol. 2022 Jul 1;3:e14. doi: 10.1017/qpb.2022.9 (PMC10095807; doi:10.1017/qpb.2022.9)
Supplement: Peer review history

# Peer review: Author comment: How alternative splicing changes the properties of plant proteins — R0/PR1

**DOI:** 10.1017/qpb.2022.9.pr1

**Authors:** Kamil Ruzicka

**Affiliations:** Laboratory of Hormonal Regulations, Institute of Experimental Botany Czech Academy of Sciences, Czech Republic

Editor Prague, 4 November 2021

Quantitative Plant Biology

Dear Editor,

Following the invitation to the Quantitative Plant Biology communicated with Dr. Olivier Hamant, please find the submission of the review article manuscript entitled How alternative splicing changes the properties of plant proteins.

The research on alternative splicing (AS) represents a dynamically expanding research field and the role of AS in plants has been repeatedly summarized from various points of view. Interestingly, none of the previous contributions attempted to conceptualize how plant AS isoforms mechanistically work, similar to the seminal review articles from the animal field,.

Respecting the uniqueness of model plant systems, we attempted to fill this gap. We gathered the prominent, well-characterized AS events that arose recently in the literature and assembled them in a similar manner as in the referred animal reviews. We thus believe that our study would serve as a comprehensive guide for any researcher interested in the general role of AS in plants.

Please see below the following experts suggested as potential referees. Though, we would be grateful for, if you do not send this manuscript to Gordon Simpson (University of Dundee, UK).

Dr. Maria Kalyna

University of Natural Resources and Life Sciences, Austria

mariya.kalyna@boku.ac.at

Expertise: molecular mechanisms of alternative splicing, transcriptomics

Assoc. Prof. Misato Ohtani

University of Tokyo, Japan

misato@edu.k.u-tokyo.ac.jp

Expertise: RNA processing in plant development

Prof. John Brown

University of Dundee, UK

John.Brown@hutton.ac.uk

Expertise: alternative splicing in plants, transcriptomics

Dr. Paula Duque

University of Lisbon, Portugal

duquep@igc.gulbenkian.pt

Expertise: alternative splicing in plants

Prof. Artur Jarmolowski

Adam Mickiewicz University, Poland

artjarmo@amu.edu.pl

Expertise: alternative splicing and RNA processing in plants

Dr. Craig Simpson

The James Hutton Institute, UK

craig.simpson@hutton.ac.uk

Expertise: mechanisms of alternative splicing in plants

Once more, we thank for the considering our contribution and the opportunity to publish in Quantitative Plant Biology.

We apologize for the delay with preparing the manuscript.

With very best wishes,

Kamil Ruzicka, Ivan Kashkan and Ksenia Timofeyenko

Institute of Experimental Botany

Czech Academy of Sciences

Rozvojová 263

165 02 Praha 6 - Lysolaje

Czechia

# Peer review: Review: How alternative splicing changes the properties of plant proteins — R0/PR2

**DOI:** 10.1017/qpb.2022.9.pr2

**Affiliations:** Technical University of Munich School of Life Sciences Weihenstephan, Germany

*Comments to Author*: This review attempts to give a comprehensive summary related to alternative splicing (AS) in plants. This is a timely and meaningful work which can be of interest to the readers of QPB. Overall the review is well written and the structure is clear. I have, however, few main comments aiming to make the review more accessible to a wide audience. A major missing part is some basic descriptions of AS in plants for non-initiated readers who lack knowledge about AS. While the author described a lot studies in details, most sections lack a clear summary and take home message. In the present form, this makes it difficult for readers to capture the key points of each section.

In the following, I provide several broad areas to improve this review article towards being more reader friendly.

1. The concept of alternative splicing

This review directly expounds the impact of AS on the transcriptome at the beginning. But it would be nice to provide an accurate definition of AS. Although biologists are generally familiar with alternative splicing, definitions and general mechanisms would still be necessary for the initial part of the review (even if citing authoritative reviews on AS as done in the introduction). Accurate descriptions can help beginners to understand alternative splicing very well.

2. Types (Modes) and mechanisms of alternative splicing

Following on the description of AS; it would be nice to see a description of the assembly mechanism of alternative splicing in the process of gene expression. The authors could first introduce the concept of AS, and then describe how alternative splicing is spliced. My suggestion is to describe the assembly process of AS according to the different assembly modes of AS (ie. exon skipping, mutually exclusive exons, alternative donor site, and so on), and draw a schematic diagram for each AS mode, which can help readers understand the process of AS more clearly.

3. The author's summary is missing in each section

To make the review go beyond the description of previous work on AS, it would be nice to have a summary of these studies combined with your own understanding as take home message. I absolutely believe that the authors have read a lot of articles to prepare this review. However, a summary of each section is lacking. This could be done also by including some future research prospects for each section.

4. Tissue-specific roles of splice isoforms

The section 2 (Diverse tissue-specific roles of splice isoforms) introduces the tissue specificity of splice isoforms. The author only describes the specificity of many splice isoforms in floral organs, I think that splice isoforms in other tissues or organs ( for example in root system(Li et al. 2016)) could also be introduced in this section.

5. Alternative splicing under stressful conditions

Adaptation of plant for stressful condition (ie. cold, drought and radiation) is one of the focal points (also include defense against biotic stress). There is big interest about roles of AS under different conditions for botanists/experimentalists. Some more general overview of research on this aspect (Li et al. 2013, Yang et al. 2014, Shang et al. 2017, Punzo et al. 2020) could be included to generalize the end of section 7. This section lists already some studies related to roles of AS under light and heat stimulation, but could include or generalize to other stresses.

6. I could suggest that the author integrate together the section 3 and section 4 (subcellular localization), as well as section 5 and section 6 (interaction between splice isoforms).

7. Overall the The conclusions are somehow redundant with each section and could be better and not refined and include a view of the current limitations in AS studies and and prospect for future research and future questions in the field.. A list of the 5 most outstanding questions in the field of plant AS could for example be included as outlook in the conclusion.

# Peer review: Review: How alternative splicing changes the properties of plant proteins — R0/PR3

**DOI:** 10.1017/qpb.2022.9.pr3

*Comments to Author*: Kashkan et al. reviewed the effects of alternative splicing (AS) on protein functionality, based on the experimental evidences. I of course agree with the importance of AS to regulate plant cell activity/function through changes in protein function/localization/activity, and this review article should be important to make the readers aware the diversified roles of AS-based protein regulation. However, unfortunately, the current version of manuscript is simply “a list of examples”. There is no clear and original perspective of advantages and/or physiological significance of AS-mediated regulation of protein functions in plants, I felt.

1. For making this review article more attractive for general readers, I suggest to reconstruct the examples of AS: in the current manuscript, the description was sometimes mixed and jumped, between molecular effects on proteins and physiological aspects, such as stress response (response to exogenous stimuli). It is so confusing; the cases shown in section 7 can be also mentioned in section 2-6, for example.

Could you re-organize the examples of AS genes/proteins perfectly based on the molecular effects? I think the best way is to follow your graphic abstract as the systematic grouping of cases for effects of AS on proteins. Or, more simply, you can insert a systematic (logical) view of molecular effect by AS (based on graphic abstract) as a summary, in the beginning of manuscript (just after Introduction). It helps the readers to understand your concept more clearly.

2. Please put the discussion on physiological significance of AS-mediated regulation of protein functions, before (or within) the section of Conclusion. No original perspective was given in your manuscript. In the discussion, you can also mention the hidden effects of AS for changing protein functionality, such as translational efficiency, mRNA stability, and mRNA localization, and which parts are possible “plant-specific aspects”.

3. In the abstract, you said “but the common underlying functional principles have surprisingly never been generalized.” What did you mean by “common underlying functional principles”? I think 1) the mode of action for AS is well studied, and its basis is widely common among eukaryotes (i.e. specific trans- and cis-factors can act as regulators), and 2) the resultant changes in protein structures/localization by AS are well summarized already; for example, the differential protein structure can change protein functionality apparently. The complicated circuits of AS regulation including splicing factors are also well known in the sex determination of insects. It should be better to make clear what is “the common underlying functional principles” in this review.

4. In Figure 1: the information in the “Mechanism” column was not well unified. For example, “tissue specificity” is not actually mechanism and not at the same level with “subcellular localization.” Rather, “tissue specificity” should be put in the same line with “temperature sensitive ” and “light intensity sensitive”, as their responsiveness. As well, you have to spell out NLS in the caption.

# Peer review: Recommendation: How alternative splicing changes the properties of plant proteins — R0/PR4

**DOI:** 10.1017/qpb.2022.9.pr4

**Affiliations:** Life Science Systems, Technische Universität München, Germany

*Comments to Author*: Dear Dr Růžička,

Many thanks for submitting to Quantitative Plant Biology.

Your review article addresses an important and interesting topic for readers of QPB, however the reviewers have concerns on the aims, structure and organization of this review article. I recommend therefore the submisison of new revised version.

After reading the review article myself I concur with the reviewers that the article at the moment reads somehow as a list of examples but clear links between them or clear take home messages are still lacking. The article could be improved by reshaping some sections and by making the structure clearer (see detailed comments by the reviewers), for example by having a first section on the bases and mechanisms of AS in plants. From there you could highlight what the main questions in the field currently are, this would yield your graphical abstract. I find that the graphical abstract is a good idea but it is not optimally used, while it could be nicely used to guide non-expert readers. This section would lay down a clear plan for the next sections. Each section would consist of several examples and a clear take home message.

I would suggest to add a “Contribution of AS to quantitative biology” section before the outlook/conclusion to show the relevance of AS links for quantitative plant biology (the aim of the journal). When reading the article I got curious about the role and importance of AS in linking genomics and phenotypes. For example what is the relevance of AS for quantitative traits (important for plant breeding), for QTL mapping/GWAs, and for allele specific expression (for important phenotypes such as abiotic stress tolerance, yield, heterosis effects). This section would allow to generalize why AS is important for crop breeding or plant evolution in general. At present most examples seem to stem from A. thaliana, but it is not always clearly stated. Maybe this section would highlight the relevance of AS in crops or in evolution of plant species in general.

The final conclusion/outlook section could highlight few major unanswered questions in the field that need to be addressed with the increasing number of large genomic, transcriptomic, metabolomic, proteomic and phenotypic data. This is one of the main aim of QPB and it would be great to get a sense of what the authors consider important for the future of big data analysis to tackle the relevance/importance/mechanisms of AS in plants.

Many thanks for submitting your work to QPB, and we look forward to receive a revised version of your article.

Sincerely,

Prof. Aurélien Tellier

# Peer review: Decision: How alternative splicing changes the properties of plant proteins — R0/PR5

**DOI:** 10.1017/qpb.2022.9.pr5

**Affiliations:** RDP, ENS de Lyon, France

No accompanying comment.

# Peer review: Author comment: How alternative splicing changes the properties of plant proteins — R1/PR6

**DOI:** 10.1017/qpb.2022.9.pr6

**Authors:** Kamil Ruzicka

**Affiliations:** Laboratory of Hormonal Regulations, Institute of Experimental Botany Czech Academy of Sciences, Czech Republic

11 March 2022

Dear Aurélien and Olivier,

Please find enclosed our revised manuscript entitled How alternative splicing changes the properties of plant proteins (QPB-21-0060).

We have discussed the points raised and revised our manuscript accordingly. We believe that the current form of the manuscript meets better the journal scope and appears to be more attractive for potential readers. The detailed response to the editor and referees’ comments is attached.

We greatly appreciate the interest of Quantitative Plant Biology in our work. We also thank the editorial team for the generousity in conceiving the revision of the manuscript and the kindly granted increase of the word limit.

With very best wishes,

Kamil Ruzicka, Ivan Kashkan and Ksenia Timofeyenko

Institute of Experimental Botany

Czech Academy of Sciences

Rozvojová 263

165 02 Praha 6 - Lysolaje

Czechia

# Peer review: Review: How alternative splicing changes the properties of plant proteins — R1/PR7

**DOI:** 10.1017/qpb.2022.9.pr7

**Affiliations:** Technical University of Munich School of Life Sciences Weihenstephan, Germany

*Comments to Author*: The authors have addressed my comments, I thus recommend the acceptance of this manuscript for the publication after a few small changes.

Although the authors provide full names for some important AS isoforms or genes, there are still many genes that have only gene symbols, not full names. It would be easy to read and check if the authors provide a table including the full and abbreviated names of all AS isoforms mentioned in this manuscript.

It would be more precise for two points as follow:

Line 60. different change to differential

Line 383. tissue change to tissue-specific

# Peer review: Review: How alternative splicing changes the properties of plant proteins — R1/PR8

**DOI:** 10.1017/qpb.2022.9.pr8

*Comments to Author*: The revised manuscript seemed to be improved, and now it better matches what QPB is looking for in a review article. Before acceptance, I would like to ask 2 minor points,

1. The authors are using “Arabidopsis” to refer to Arabidopsis thaliana. AS events can vary widely among plant species, so in my sense, the authors should clearly limit the specie to Arabidopsis thaliana. At least when it first appears, please write the species name as “Arabidopsis thaliana.”

2. Please mention about “exitron” in the introduction briefly; it is very important form of intron, and it has been shown that the high rate of exitron is a very unique feature of plants as you know. It unnatural that there is no mention of exitron when discussing AS in plants.

# Peer review: Recommendation: How alternative splicing changes the properties of plant proteins — R1/PR9

**DOI:** 10.1017/qpb.2022.9.pr9

**Affiliations:** Life Science Systems, Technische Universität München, Germany

*Comments to Author*:

Dear authors,

I apologize for the time to get the reviews in this round. I concur with the reviewers that the paper has been greatly improved, and gained a lot in clarity. Both reviewers recommend publication pending some last minor changes.

Reviewer 1 suggests to make sure that full names of genes/isoforms are written somewhere (in text or table) and not only the gene symbols.

Reviewer 2 suggests to be precise when to use Arabidopsis or Arabidopsis thaliana, and to mention exitron briefly in the introduction.

Providing you address these minor changes, the manuscript will be accepted for publication.

I look forward to receive your revised version soon.

Best regards,

Aurelien Tellier

# Peer review: Decision: How alternative splicing changes the properties of plant proteins — R1/PR10

**DOI:** 10.1017/qpb.2022.9.pr10

**Affiliations:** RDP, ENS de Lyon, France

No accompanying comment.

# Peer review: Author comment: How alternative splicing changes the properties of plant proteins — R2/PR11

**DOI:** 10.1017/qpb.2022.9.pr11

**Authors:** Kamil Ruzicka

**Affiliations:** Laboratory of Hormonal Regulations, Institute of Experimental Botany Czech Academy of Sciences, Czech Republic

Dear Aurélien and Olivier,

Please find enclosed our second revision of the manuscript How alternative splicing changes the properties of plant proteins (QPB-21-0060). We have corrected the remaining minor points as requested.

We thank you for the opportunity to contribute to QPB and for the helpful editorial and reviewer comments. We hope our work will be of interest to many prospective readers!

With very best wishes,

Kamil Ruzicka, Ivan Kashkan and Ksenia Timofeyenko

Laboratory of Hormonal Regulations in Plants

Prague, 29 April 2022

# Peer review: Recommendation: How alternative splicing changes the properties of plant proteins — R2/PR12

**DOI:** 10.1017/qpb.2022.9.pr12

**Affiliations:** Life Science Systems, Technische Universität München, Germany

No accompanying comment.

# Peer review: Decision: How alternative splicing changes the properties of plant proteins — R2/PR13

**DOI:** 10.1017/qpb.2022.9.pr13

**Affiliations:** RDP, ENS de Lyon, France

No accompanying comment.
